# Carpal tunnel syndrome with both AA amyloidosis and elderly-onset Still disease: A case report

**DOI:** 10.1097/MD.0000000000044327

**Published:** 2025-09-05

**Authors:** Osamu Nakamura, Yoichi Ishibashi, Kentaro Ono, Tatsuro Ogata, Masakazu Ishikawa

**Affiliations:** a Department of Orthopedic Surgery, Kagawa Prefectural Shirotori Hospital, Higashikagawa City, Japan; b Department of Cardiovascular Medicine, Kagawa Prefectural Shirotori Hospital, Higashikagawa City, Japan; c Department of Orthopedic Surgery, Faculty of Medicine, Kagawa University, Kagawa, Japan.

**Keywords:** AA amyloidosis, adult-onset Still disease, carpal tunnel syndrome

## Abstract

**Rationale:**

This study reports a rare case of both AA amyloidosis and elderly-onset Still disease presenting as fever following carpal tunnel syndrome surgery.

**Patient concerns:**

A 79-year-old man reported numbness, pain, and muscle weakness in his right hand for several months.

**Diagnoses:**

We performed carpal tunnel opening surgery and a synovial biopsy because of significant synovial tissue in the carpal tunnel. A pathological examination revealed amyloid tissue deposition in the interstitium around the blood vessels and between the muscle bundles. He developed a postoperative fever. Blood biochemical tests revealed elevated neutrophils and blood sedimentation as well as high C-reactive protein, serum amyloid A protein, and ferritin levels. He was diagnosed with adult Still disease, and the carpal tunnel syndrome was suspected to have resulted from AA amyloidosis.

**Interventions:**

With steroid pulse therapy, his inflammatory response, fever, joint pain, and swelling rapidly improved.

**Outcomes:**

In the first year postoperative, the clinical findings improved and patient-reported outcomes were good.

**Lessons:**

Elderly-onset Still disease presents as an unknown fever after carpal tunnel release surgery and is likely caused by AA amyloidosis in older men. Here, we detailed a rare case of both AA amyloidosis and adult-onset Still disease diagnosed of the carpal tunnel’s synovial membrane.

## 1. Introduction

Adult-onset Still disease (AOSD) is a systemic inflammatory disorder of unknown etiology characterized by a high-spiking fever, evanescent rash, polyarthritis, and many other manifestations including odynophagia, lymphadenopathy, splenomegaly, hepatic and pulmonary involvement, and serositis.^[1]^ AOSD, first described by Bywater in 1971,^[2]^ primarily affects young individuals, with a bimodal age distribution peaking at 15 to 25 and 36 to 46 years at disease onset.^[3,4]^ Although AOSD is less common in older individuals, some cases of elderly-onset Still disease (EOSD) have been reported.^[5–7]^

AA amyloidosis (AAA) is a multisystemic disease associated with serum amyloid A (SAA) protein deposition in tissues that occurs secondary to chronic inflammation. AAA is caused by multiple factors including chronic rheumatic and inflammatory bowel diseases, monogenic autoinflammatory diseases, chronic infections, less frequent cancers, and immune deficiencies. Furthermore, recurrent or persistent AOSD with suboptimal disease control is associated with multiple complications such as chronic destructive arthritis, increased long-term morbidity and mortality rates, and AAA.^[8]^ Here we report a rare case of EOSD presenting as an unknown fever following surgery for carpal tunnel syndrome that was thought to be caused with AAA in an older man.

## 2. Case presentation

**Figure 1.**
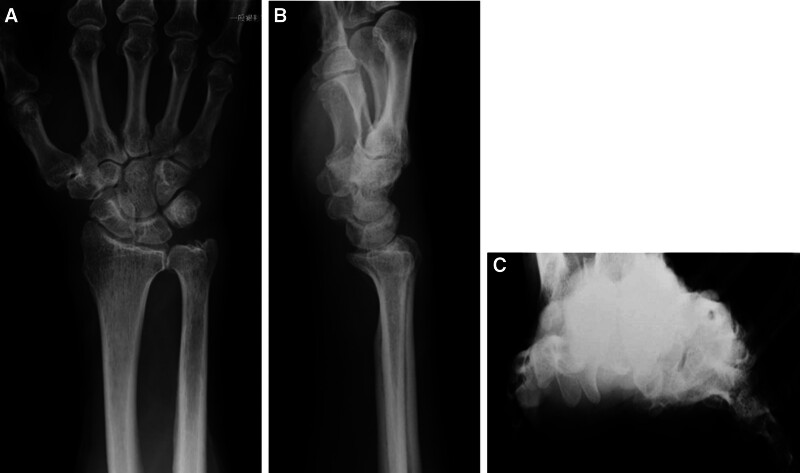
The radiology images show the certification in carpal tunnel. (A) AP view; (B) lateral view; (C) carpal tunnel view.

**Figure 2.**
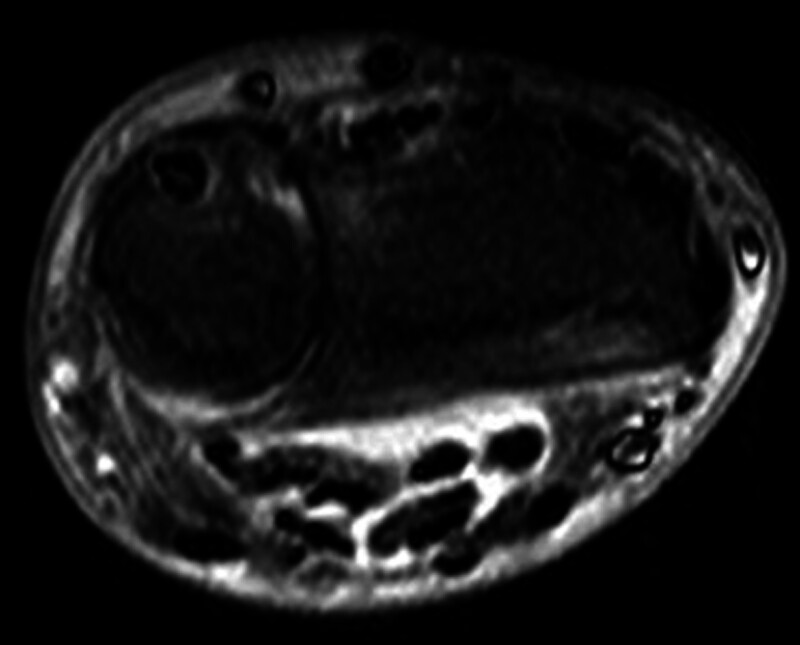
T2-weighted magnetic resonance imaging showing a high degree of synovial growth inside the carpal tunnel.

**Figure 3.**
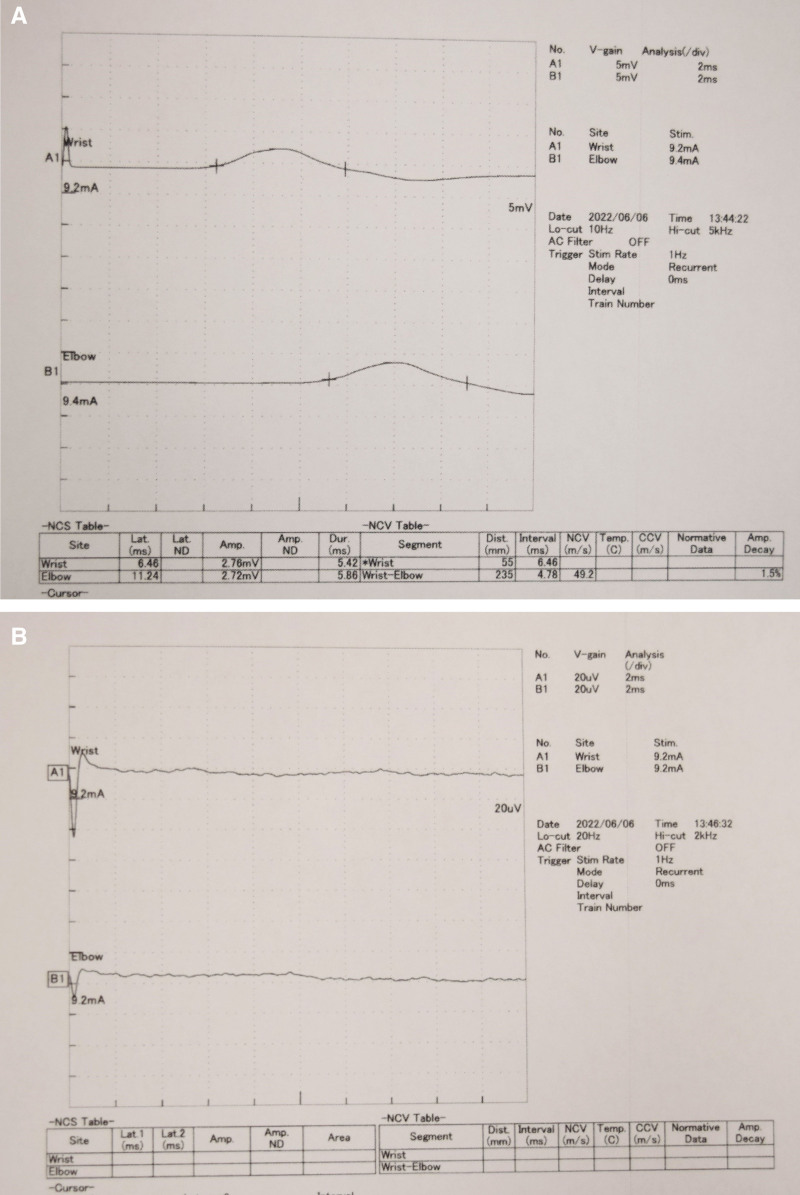
An electrophysiological evaluation before operation. (A) Latent prolongation of the median nerve (6.46 ms); (B) no response elicited by sensory nerve stimulation.

**Figure 4.**
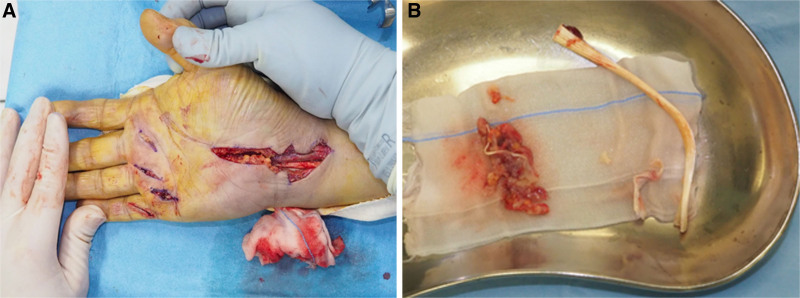
Intraoperative findings. (A) Tendon sheath incisions made in the middle, ring, and little fingers; (B) excision of the flexor digitorum superficialis tendon of the ring finger with a marked flexion contracture.

**Figure 5.**
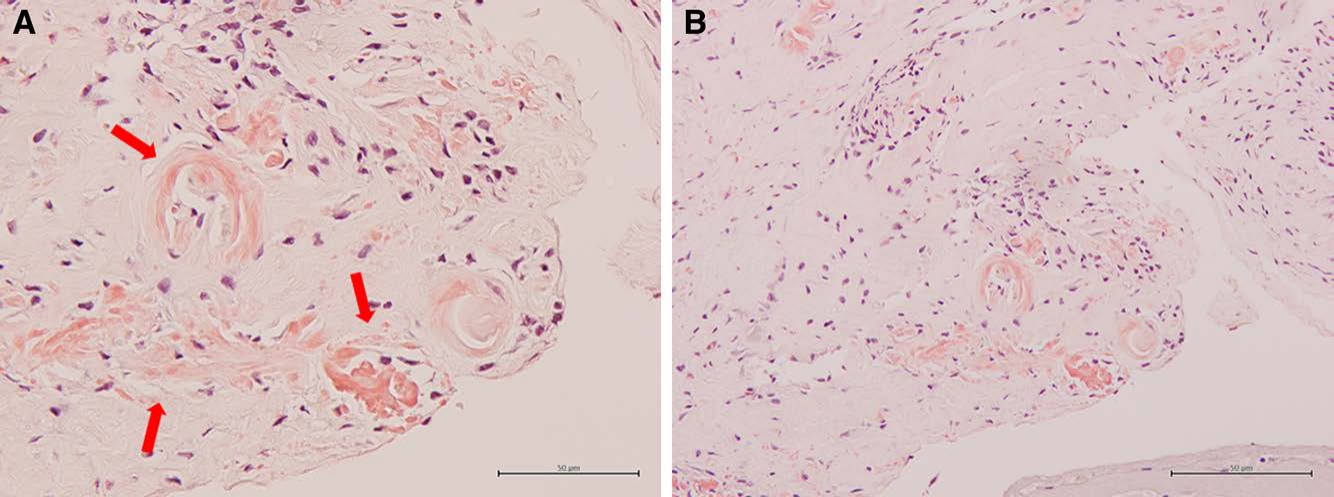
Photomicrograph of the resected synovial tissue showing amyloid deposition in the interstitium around the blood vessels and between the muscle bundles (hematoxylin and eosin staining; [A] high-power view; [B] low-power view).

A 79-year-old man, who had a history of type 2 diabetes mellitus and experienced numbness, pain, and muscle weakness in his right hand for several months without any identifiable triggers, was referred to our hospital in March 2021. He was very disappointed that he could not play his hobby of table tennis because of the symptoms in his right hand. His right wrist joint was swollen, and a physical examination revealed decreased grip (17.4 kg [right], 28.6 kg [left]) and pinching strength (5 kg [right], 6 kg [left]), thumb muscle atrophy on inspection, thumb opposition dysfunction, nocturnal pain, and flexor tendinitis and snapping phenomenon of the middle, ring, and little fingers on palpation. Motor examination revealed atrophy of thenar muscles and weakness of thumb abduction. Flexor tendinitis was diagnosed based on the medical history and physical examinations that were tenderness over the flexor tendon sheath, chronic flexion contracture of the proximal interphalangeal joint, painful snapping during flexion and extension movements. In particular, the ring finger had significant flexion contracture with extension restriction of −30° or more. Radiography revealed carpal tunnel calcification (Fig. 1). Magnetic resonance imaging revealed a high degree of synovial growth within the carpal tunnel (Fig. 2). An electrophysiological evaluation revealed latent prolongation of the median nerve (6.46 ms) with no response elicited by sensory nerve stimulation (Fig. 3). We diagnosed the patient with carpal tunnel syndrome and performed release surgery along with a synovial biopsy because of a significant increase in synovial tissue within the carpal tunnel. Simultaneously, incisions were made in the tendon sheaths of the middle, ring, and little fingers, and the flexor digitorum superficialis tendon of the ring finger, which exhibited a marked flexion contracture, was excised (Fig. 4). A histological examination of the resected synovial tissue revealed amyloid deposition in the interstitium around the blood vessels and between the muscle bundles (Fig. 5). After the surgery, he wore the cock-up splint of wrist for a week. At the same time, active exercises of tendon gliding, and passive stretching of the flexor tendons were began. These rehabilitations were continued for 3 months after surgery.

**Figure 6.**
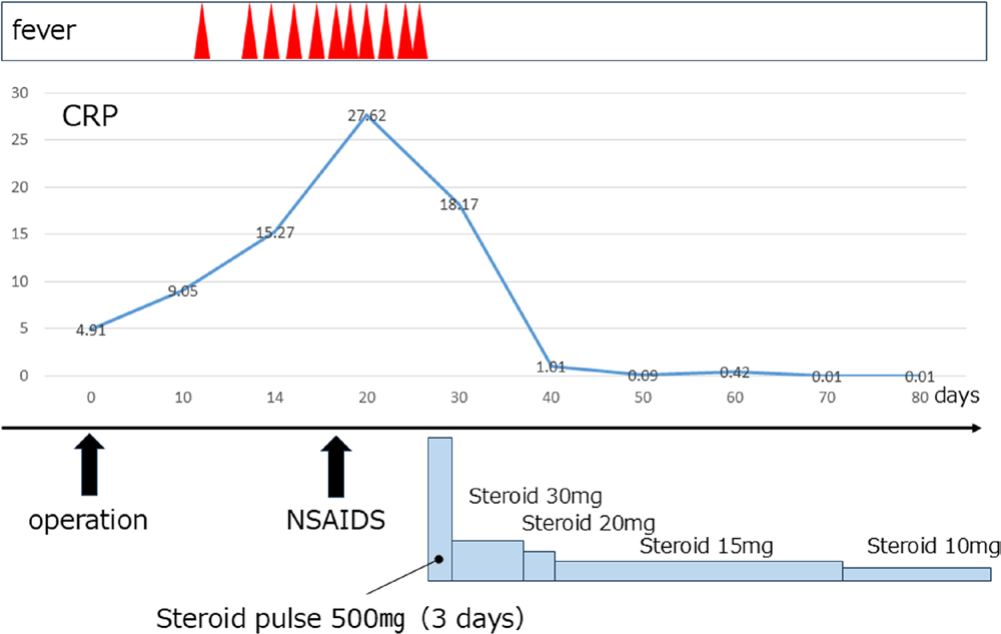
Patient’s clinical course.

**Figure 7.**
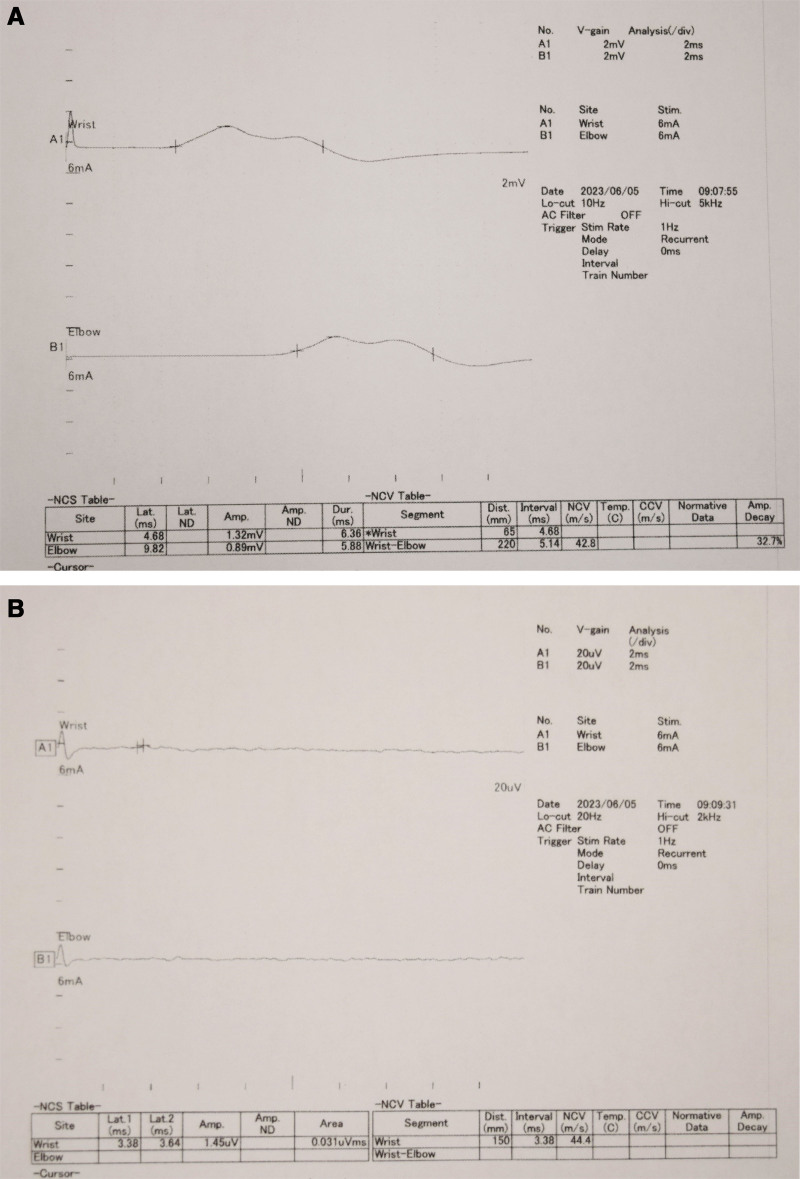
An electrophysiological evaluation after operation. (A) Latent prolongation of the median nerve (4.68 ms); (B) the evaluation of the sensory nerve by median nerve stimulation allowed for slight amplitude measurement.

Postoperatively, the patient spiked a fever that lasted for several days. Blood biochemical tests revealed abnormally high levels of inflammatory markers. He was given NSAIDS but his fever did not subside. An endoscopic gastric biopsy tissue examination revealed gastric mucosal tissue in the fundic gland region, with mild inflammatory cell infiltration, mainly lymphocytes, but no amyloid deposits or heart failure; therefore, systemic amyloidosis was considered negative. Subsequent blood biochemical tests revealed an elevated neutrophilic white blood cell count and blood sedimentation as well as high levels of C-reactive protein, SAA, ferritin, and ESR. However, the antinuclear antibody levels were normal (Table 1). In addition, the patient had bilateral wrist joint arthritis and a salmon-pink skin rash. Notably, the patient was diagnosed with AOSD based on the classification criteria of Yamaguchi et al,^[9]^ and the secondary carpal tunnel syndrome was considered caused with AAA. Accordingly, after intravenous methylprednisolone (Solu-Medrol) steroid pulse therapy (500 mg 3 days), steroid (oral prednisone) decreased with tapered doses of 30 mg 14 days, 20 mg 5 days, 15 mg 30 days, 10mg 1 year and 7.5 mg led to rapid improvement in the inflammatory blood examination indices, fever, joint pain, and swelling (Fig. 6). In the first year postoperative, the clinical findings improved. Specifically, grip power (22.9 kg [right], 27.9 kg [left]), pinching strength (6.5 kg [right], 7.0 kg [left]) were increased, and patient-reported outcomes were good, 2.27 points in QuickDASH Disability/Symptom scoring and 3.16 points in Hand20 scoring. The electrophysiological values improved, latent prolongation of the median nerve (4.68 ms) and the evaluation of the sensory nerve by median nerve stimulation allowed for slight amplitude measurement (Fig. 7).

**Table 1:** Blood biochemical test results.

WBC	111 × 10^2^/μL	Antinuclear antibody	<40
RBC	430 × 10^4^/μL	ESR-1H	111 mm
Hb	12.7 g/dL	BUN	17.2 mg/dL
PLT	30.9 × 10^2^/dL	eGFR	36.9
Baso (D)	0.2%	UA	5.6 mg/dL
Eos (D)	0.4%	MMP-3	149.0 ng/dL
Neut (D)	79.3%	β2MG	3.0
Lymp (D)	13.9%	Amyloid A	633.5 μg/mL
Mono (D)	6.2%	Rheumatoid factor	<3 U/mL
CRP	27.62 mg/dL	Ferritin	366.0 ng/mL
AST	33 IU/dL		
ALT	45 IU/dL		

## 3. Discussion

AOSD is clinically characterized by a high fever, polyarthralgia, and skin rashes. Specifically, the fever pattern is characterized by a spike-shaped heat generation as in this case. A peripheral blood examination of patients with AOSD often reveals significantly higher inflammatory responses, with a negative antinuclear antibody result and elevated serum ferritin level^[10]^ as in the present case. Complications of AOSD such as disseminated intravascular coagulation, interstitial pneumonia, and rare secondary amyloidosis have been reported.^[1]^ The mean age of patients with typical AOSD is 36 years,^[11]^ lower than that of the patient in this case. However, some cases of EOSD have been reported previously.^[6,7]^

The pathophysiology of AOSD remains unclear. However, factors such as an imbalance in innate and adaptive immunity and increased inflammatory cytokine levels contribute to its development.^[12,13]^ In this case, AOSD with rapid deterioration (wrist joint swelling) was considered triggered by the surgical treatment.

In the past, treating AOSD was challenging because of the limited therapeutic options for nonsteroidal anti-inflammatory drugs, glucocorticoids, and conventional synthetic disease-modifying antirheumatic drugs. Glucocorticoids remain the mainstay of treatment for AOSD; however, disease-modifying antirheumatic drugs are often required in patients who respond poorly to glucocorticoids. The advent of targeted biological treatments for rheumatic diseases has revolutionized the management of AOSD, especially its refractory forms.^[14]^

In a report of secondary AAA in AOSD, the amyloid load increased and organ function deteriorated in most patients whose SAA level was persistently above 50 mg/L. SAA is a prognostic marker for AAA, with favorable outcomes associated with serum concentrations of <10 mg/L. The diagnosis of amyloidosis was confirmed through renal, intestinal bladder, abdominal fat, and salivary gland biopsies, with pathology reports typically indicating vascular and perivascular amyloid depositions.^[1,15]^ In this case, amyloid deposition was not detected on a gastric biopsy; however, amyloid deposits were detected on a carpal tunnel synovial tissue biopsy.

Sugiura et al reported that 34% of the patients who underwent carpal tunnel release surgery had transthyretin amyloid deposition in the tenosynovial tissue.^[16]^ Notably, reports of carpal tunnel syndrome complicated by secondary amyloidosis with AOSD were not included in our investigation. Therefore, this was a rare case of adult styloidosis diagnosed as AAA of the carpal tunnel synovial membrane. In the future, as noted in other reports, AOSD may develop in organs such as the kidneys, bladder, and stomach; therefore, further follow-up is necessary.

Here we reported a case of EOSD presenting as an unknown fever following carpal tunnel release surgery that was considered caused with AAA in an older man. This was a rare case of AOSD diagnosed as amyloidosis of the carpal tunnel synovial membrane. Although a histological examination is necessary for a definitive diagnosis, EOSD should be included in the differential diagnosis of multiple arthritic lesions with a high-spiking fever in older patients.

## Acknowledgments

We would like to thank *Editage* (www.editage.com) for English language editing.

## Author contributions

**Resources:** Tatsuro Ogata.

**Supervision:** Masakazu Ishikawa.

**Writing – original draft:** Osamu Nakamura.

**Writing – review & editing:** Yoichi Ishibashi, Kentaro Ono.
